# Complete mitochondrial genome of *Striatobalanus tenuis* Hoek, 1883 (Balanomorpha: Balanidae) and a novel molecular phylogeny within Cirripedia

**DOI:** 10.1080/23802359.2023.2299087

**Published:** 2024-01-03

**Authors:** Ning Mao, Wentai Shao, Sheng Mao, Yuefeng Cai, Nanjing Ji, Xin Shen

**Affiliations:** a Jiangsu Institute of Marine Resources/Jiangsu Key Laboratory of Marine Biotechnology, Jiangsu Ocean University, Lianyungang, China; b Co-Innovation Center of Jiangsu Marine Bio-industry Technology, Jiangsu Ocean University, Lianyungang, China

**Keywords:** *Striatobalanus tenuis*, balanomorpha, mitogenome, phylogeny

## Abstract

Barnacles are crustaceans that are critical model organisms in intertidal ecology and biofouling research. In this study, we present the first mitochondrial genome of *Striatobalanus tenuis* which is a circular molecule of 15,067 bp in length. Consistent with most barnacles, the mitochondrial genome of *S. tenuis* encodes 37 genes, including 13 PCGs, 22 tRNAs and 2 rRNAs. A novel insight into the phylogenetic analysis based on the nucleotide data of 13 PCGs showed that the *S. tenuis* clusters with *Striatobalanus amaryllis* (bootstrap value = 100) of the same genus, then groups with other Balanoidea species, the Chelonibiidae, Austrobalanidae and Tetraclitidae cluster together forming superfamily Coronuloidea. The result can help us to understand the novel classification within Balanomorpha.

## 1. Introduction

Cirripedia are sessile crustaceans which inhabit marine environments ranging from the intertidal to the deep-sea, with certain species exhibiting parasitic behavior toward marine organisms (Chan et al. 2021). These organisms hold significant ecological and economic value, thus attracting extensive research attention in the fields of developmental biology, crustacean evolution, and ecotoxicology (Yu and Chan 2020; Zhao et al. 2022). The barnacle *Striatobalanus tenuis* Hoek, 1883 inhabits tropical, subtropical and temperate subtidal zones. In China, it primarily resides offshore in the Southeast China Sea, typically attaching itself to shells, stones, stony corals and occasionally the cephalothorax of crabs. Liu (2013) discovered that as a common inhabitant in the coastal waters, *S. tenuis* occupies an important place among the fouling acorn barnacle community. *S. tenuis* belongs to the genus Striatobalanus which has eight species (Chan et al. 2021). To elucidate the phylogenetic relationship within the order Balanomorpha, we sequenced and analyzed the mitogenome of *S. tenuis*. This study will not only present the first mitogenome of *S. tenuis*, but will also provide a fundamental basis for future investigations of phylogenetic relationships within the order Balanomorpha.

## 2. Materials and methods

**Figure 1.**
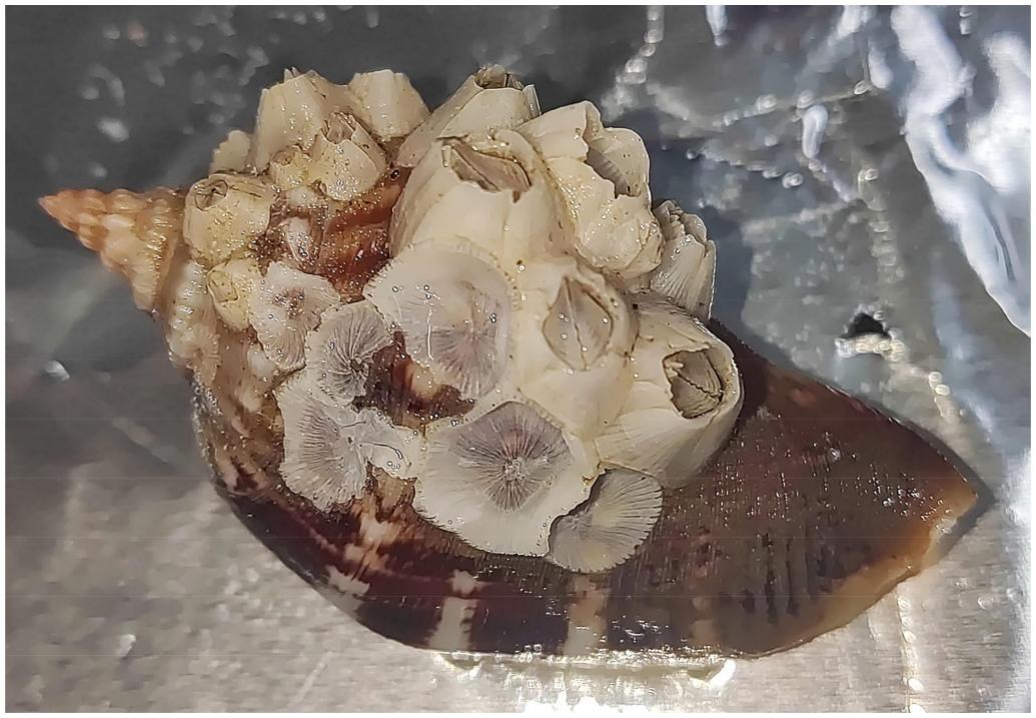
The image of *Striatobalanus tenuis* Hoek, 1883. It was taken by ning Mao in our laboratory.

In this study, the specimens of *S. tenuis* were collected from a hermit crab in Xiamen (24.44°N, 118.08°E), Fujian Province, China. *S. tenuis* specimen was deposited at the Marine Museum of Jiangsu Ocean University (https://sea.jou.edu.cn/, Yuefeng Cai, and yuefengcai@ jou.edu.cn) under the voucher number Sten-001 (Figure 1). The muscle tissue isolated from the fresh specimen was immediately preserved in 95% ethanol. Subsequently, the total DNA was extracted from the muscle tissue using the TIANamp DNA Kit (TIANGEN, Beijing, China) according to the manufacturer’s instructions. The DNA was then randomly fragmented into 350 bp fragments, followed by end repair. These fragments were amplified through PCR to construct a sequencing library, which was ultimately sequenced on the Illumina NovaSeq 6000 platform (TSINGKE Biotechnology Co, Ltd, China). Sequence quality control was conducted to acquire clean reads, which were subsequently assembled using SPAdes 3.13.0 (Bankevich et al. 2012). The assembly results were further supplemented by Gapcloser software. The assembled mitogenomes were presented in Supplementary Figure S1 and Table S4. Gene annotation was conducted by the online MITOS tool (http://mitos.bioinf.unileipzig.de) and tRNAscan-SE (Chan and Lowe 2019). A maximum-likelihood phylogenetic tree was constructed using the bootstrap procedure with 1,000 replications as implemented based on 13 protein-coding genes (PCGs) of *S. tenuis* and other barnacles from Balanomorpha in PhyloSuite (Zhang et al. 2020).

## 3. Results

**Figure 2.**
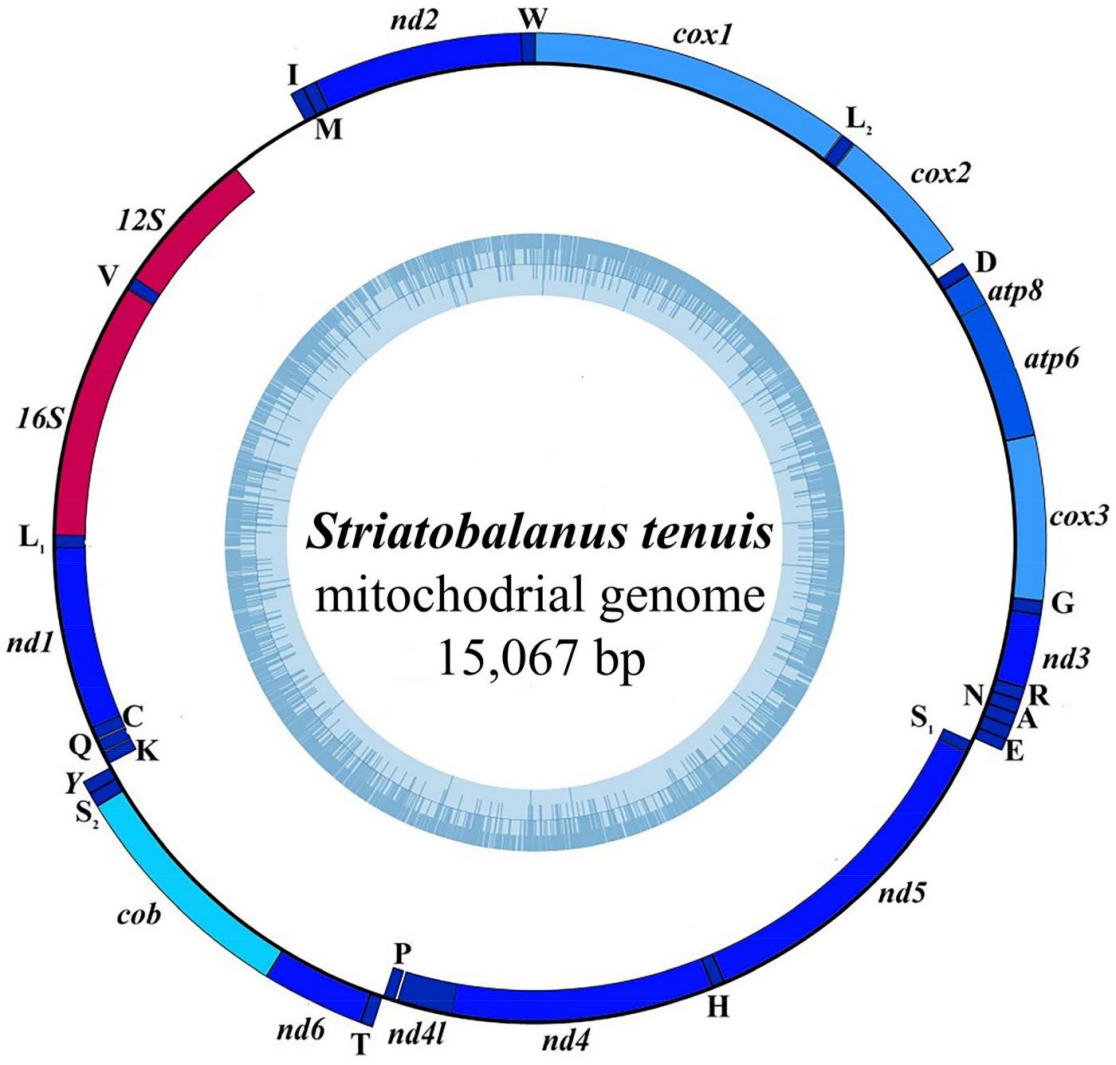
The complete mitogenomes map of *Striatobalanus tenuis* Hoek, 1883. The heavy strand is represented by the outer circle, while the light strand is represented by the inner circle; the tRNA and PCGs are denoted by the blue genes, whereas the 12sRNA and 16sRNA are indicated by the red genes.

The complete mitogenomes of *S. tenuis* is a circular DNA molecule containing 15,067 bp, which encodes 13 PCGs, 22 tRNA genes and 2 rRNA genes (GenBank accession number: OL979158) (Figure 2 and Supplementary Table S1). The base composition of *S. tenuis* mitogenome is 37.4% A, 16.2% C, 11.1% G and 35.3% T with a higher A + T value (72.8%) (Supplementary Table S2). The length of PCGs is 11,050 bp (73.3%), which is consistent with other available mitochondrial genomes of Balanidae. All non-coding regions are 561 bp in length, and the longest one (417 bp) is located between *srRNA* and *trnI*. The start codon for all PCGs in *S. tenuis* are ATG, ATT and ATA. Notably, *cox3*, *nd3* and *nd5* are terminated by the codon T-, while the remaining PCGs have the complete terminator codon TAA or TAG. The A + T content of *srRNA* is 69.9%, while *lrRNA* has a content of 77%. The *S. tenuis* genome contains 3,669 codons (excluding incomplete terminator codons), across its 13 PCGs, with leucine being the most frequently used amino acid is leucine (549), followed by phenylalanine (364), serine (332), and isoleucine (330) (Supplementary Table S3).

## 4. Discussion and conclusion

**Figure 3.**
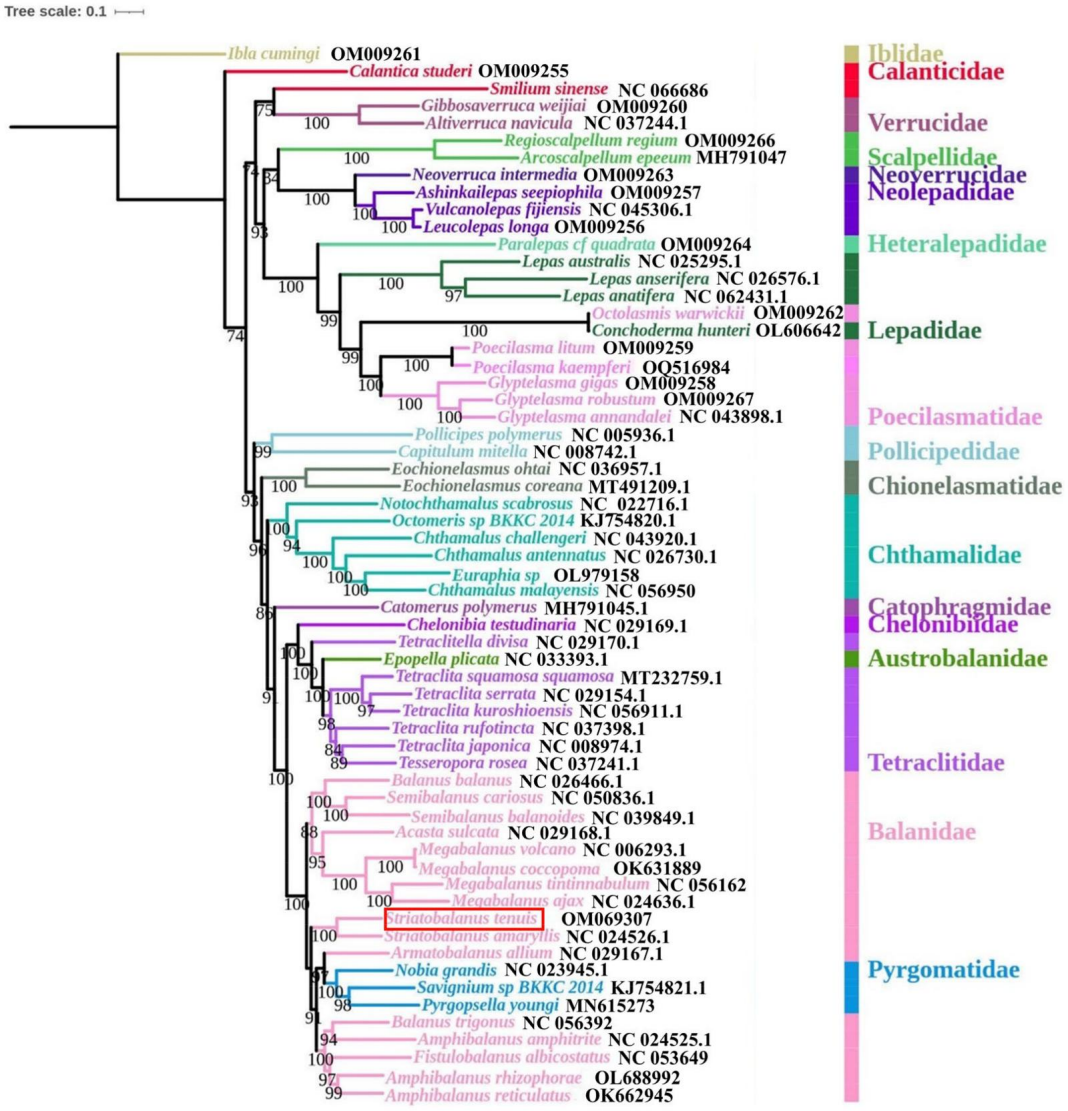
The maximum-likelihood phylogenetic tree based on 13 PCGs of *Striatobalanus tenuis* and other mitogenomes from Balanomorpha with barnacle *Ibia cumingi* as outgroups. Red box represents *Striatobalanus tenuis* in this study. Different colors indicate different barnacle families.

The selection pressures exerted by habitat environments on genes necessitate further investigation into the impact of base migration on the initiation or direction of gene replication, as well as the identification of the selection pressures that contribute to the occurrence of migration. Additionally, the utilization of mitochondrial codons can influence gene expression and reflect evolutionary relationships between species (Wei et al. 2014). To comprehend the connections between *S. tenuis* and the other species in Balanomorpha, a phylogenetic tree was constructed based on the nucleotide data of 13 PCGs. This dataset included all available Balanomorpha species in NCBI databases with *Ibia cumingi* as the outgroup. iTOL online website (Letunic and Bork 2016) was performed to view and beautify the phylogenetic tree (Figure 3). The phylogenetic tree constructed by mitochondrial genes exhibits a consistent topological structure and demonstrates a high rate of node support. As research on the mitochondrial genome of Balanomorpha advances, the understanding of the phylogenetic relationships among its various species is progressively clear (Zhang et al. 2023). Referring to previous research, a novel classification among Balanomorpha is used to clarify the phylogenetic relationships (Chan et al. 2021). As depicted in Figure 3, S. *tenuis* and *S. amaryllis*, belonging to the same genus, are grouped in a single branch with a bootstrap value of 100. Furthermore, these two species exhibit identical gene order. Additionally, species from the Balanoidea superfamily, namely Balanidae and Pyrgomatidae, form clusters with high bootstrap values of 100. In addition, the phylogenetic tree also shows that the Chelonibiidae, Austrobalanidae and Tetraclitidae cluster together forming the superfamily Coronuloidea. In numerous aspects, it aligns with the evolutionary results of other phylogenetic analyses (Cai et al. 2018; Chan et al. 2021; Mao et al. 2021).

This study presents the first complete mitochondrial genome of *S. tenuis*, contributing to the expanded genomic resources of species within Balanomorpha and offering valuable data to support the phylogenetic analysis of Balanomorpha.

## Supplementary Material

Supplemental Material

Supplemental Material

Supplemental Material

Supplemental Material

## Data Availability

The genome sequence data that support the findings of this study are openly available in GenBank of NCBI at https://www.ncbi.nlm.nih.gov/ under accession no. OL979158. The associated BioProject, SRA, and Bio-Sample numbers are PRJNA1005023, SRR25626966, SAMN36971048 and SAMN36971049, respectively.
