# Supplementary material for: Complete mitochondrial genome of Striatobalanus tenuis Hoek, 1883 (Balanomorpha: Balanidae) and a novel molecular phylogeny within Cirripedia
Source: Mitochondrial DNA B Resour. 2024 Jan 3;9(1):29–32. doi: 10.1080/23802359.2023.2299087 (PMC10769117; doi:10.1080/23802359.2023.2299087)
Supplement: Supplemental Material [file TMDN_A_2299087_SM5173.docx]

Table S1. The complete mitogenomes annotation of *Striatobalanus tenuis*

| Gene | Strand | Position | | Nucleotides | Codons | | Anti-codon | Intergenic sequence * |
| --- | --- | --- | --- | --- | --- | --- | --- | --- |
|  |  | Start | Stop |  | Start | Stop |  |  |
| *cox1* | H | 1 | 1545 | 1545 | ATA | TAA |  | 2 |
| *trnL_2_* | H | 1548 | 1615 | 68 |  |  | taa | 2 |
| *cox2* | H | 1618 | 2301 | 684 | ATG | TAA |  | 73 |
| *trnD* | H | 2375 | 2438 | 64 |  |  | gtc | 0 |
| *atp8* | H | 2439 | 2597 | 159 | ATT | TAA |  | -7 |
| *atp6* | H | 2591 | 3256 | 666 | ATG | TAA |  | 0 |
| *cox3* | H | 3257 | 4043 | 787 | ATG | T- |  | 0 |
| *trnG* | H | 4044 | 4108 | 65 |  |  | tcc | 0 |
| *nad3* | H | 4109 | 4460 | 352 | ATT | T- |  | 0 |
| *trnR* | H | 4461 | 4523 | 63 |  |  | tcg | 0 |
| *trnN* | H | 4524 | 4588 | 65 |  |  | gtt | 0 |
| *trnA* | H | 4589 | 4653 | 65 |  |  | tgc | 0 |
| *trnE* | H | 4654 | 4718 | 65 |  |  | ttc | 0 |
| *trnS_1_* | H | 4719 | 4776 | 58 |  |  | gct | 16 |
| *trnF* | L | 4793 | 4856 | 64 |  |  | gaa | 1 |
| *nad5* | L | 4858 | 6559 | 1702 | ATT | T- |  | 0 |
| *trnH* | L | 6560 | 6623 | 64 |  |  | gtg | 0 |
| *nad4* | L | 6624 | 7953 | 1330 | ATG | TAA |  | -7 |
| *nad4l* | L | 7947 | 8231 | 285 | ATA | TAA |  | 9 |
| *trnP* | L | 8241 | 8303 | 63 |  |  | tgg | 2 |
| *trnT* | H | 8306 | 8368 | 63 |  |  | tgt | 15 |
| *nad6* | H | 8384 | 8857 | 474 | ATT | TAA |  | -1 |
| *cob* | H | 8857 | 9996 | 1140 | ATG | TAG |  | -2 |
| *trnS_2_* | H | 9995 | 10064 | 70 |  |  | tga | 0 |
| *trnY* | H | 10065 | 10128 | 64 |  |  | gta | 18 |
| *trnK* | L | 10147 | 10210 | 64 |  |  | ttt | 2 |
| *trnQ* | L | 10213 | 10280 | 68 |  |  | ttg | 3 |
| *trnC* | L | 10284 | 10349 | 66 |  |  | gca | -2 |
| *nad1* | L | 10348 | 11274 | 927 | ATA | TAA |  | -3 |
| *trnL_1_* | L | 11272 | 11339 | 68 |  |  | tag | 0 |
| *lrRNA* | L | 11321 | 12639 | 1300 |  |  |  | 1 |
| *trnV* | L | 12641 | 12706 | 66 |  |  | tac | -5 |
| *srRNA* | L | 12702 | 13455 | 754 |  |  |  | 417 |
| *trnI* | H | 13873 | 13939 | 67 |  |  | gat | 0 |
| *trnM* | H | 13940 | 14005 | 66 |  |  | cat | 0 |
| *nad2* | H | 14006 | 15004 | 999 | ATG | TAA |  | -2 |
| *trnW* | H | 15003 | 15067 | 65 |  |  | tca | 0 |

Note: * Negative numbers indicate overlapping nucleotides between adjacent genes.

Table S2. Nucleotide composition and skew of *Striatobalanus tenuis* mitogenomes

| Gene | Proportion of nucleotides | | | | A+T  (%) | AT skew | GC skew |
| --- | --- | --- | --- | --- | --- | --- | --- |
|  | A(%) | C(%) | G(%) | T(%) |  |  |  |
| *atp6* | 32.3 | 16.1 | 10.1 | 41.6 | 73.9 | -0.126 | -0.229 |
| *atp8* | 35.9 | 18.2 | 5.7 | 40.3 | 76.1 | -0.058 | -0.524 |
| *cob* | 30.4 | 17.1 | 13.5 | 39.0 | 69.4 | -0.124 | -0.118 |
| *cox1* | 30.4 | 15.6 | 16.3 | 37.7 | 68.1 | -0.107 | 0.022 |
| *cox2* | 34.1 | 17.1 | 11.8 | 37.0 | 71.1 | -0.041 | -0.183 |
| *cox3* | 28.0 | 18.2 | 14.7 | 39.1 | 67.1 | -0.165 | -0.106 |
| *nd1* | 24.1 | 10.0 | 18.0 | 47.9 | 72.0 | -0.331 | 0.286 |
| *nd2* | 31.7 | 14.7 | 9.3 | 44.2 | 76.0 | -0.165 | -0.225 |
| *nd3* | 20.7 | 16.8 | 10.2 | 42.3 | 73.0 | -0.343 | -0.244 |
| *nd4* | 26.5 | 9.1 | 16.5 | 48.0 | 74.4 | -0.289 | 0.289 |
| *nd4L* | 30.2 | 9.5 | 17.9 | 42.5 | 72.6 | -0.169 | 0.307 |
| *nd5* | 28.32 | 10.4 | 15.4 | 45.9 | 74.2 | -0.237 | 0.194 |
| *nd6* | 35.1 | 16.9 | 5.7 | 42.1 | 77.3 | -0.091 | -0.496 |
| *srRNA* | 35.2 | 11.0 | 19.2 | 34.8 | 69.9 | 0.006 | 0.272 |
| *lrRNA* | 37.2 | 8.3 | 14.6 | 39.9 | 77.0 | -0.035 | 0.275 |
| All PCGs | 29.7 | 14.0 | 13.8 | 42.7 | 72.2 | -0.180 | -0.007 |
| All | 37.4 | 16.2 | 11.1 | 35.3 | 72.8 | 0.029 | -0.187 |

Table S3. Condon usage in *Striatobalanus tenuis*

| Condon | Number | Percent | Condon | Number | Percent | Condon | Number | Percent | Condon | Number | Percent |
| --- | --- | --- | --- | --- | --- | --- | --- | --- | --- | --- | --- |
| UUU-F | 312 | 8.48 | UCU-S | 146 | 3.97 | UAU-Y | 114 | 3.10 | UGU-C | 33 | 0.90 |
| UUC-F | 52 | 1.41 | UCC-S | 19 | 0.52 | UAC-Y | 33 | 0.90 | UGC-C | 1 | 0.03 |
| UUA-L | 343 | 9.33 | UCA-S | 97 | 2.64 | UAA-* | 0 | 0.00 | UGA-W | 92 | 2.50 |
| UUG-L | 37 | 1.01 | UCG-S | 1 | 0.03 | UAG-* | 0 | 0.00 | UGG-W | 10 | 0.27 |
| CUU-L | 82 | 2.23 | CCU-P | 79 | 2.15 | CAU-H | 52 | 1.41 | CGU-R | 29 | 0.79 |
| CUC-L | 8 | 0.22 | CCC-P | 12 | 0.33 | CAC-H | 22 | 0.60 | CGC-R | 0 | 0.00 |
| CUA-L | 73 | 1.98 | CCA-P | 50 | 1.36 | CAA-Q | 52 | 1.41 | CGA-R | 28 | 0.76 |
| CUG-L | 6 | 0.16 | CCG-P | 1 | 0.03 | CAG-Q | 11 | 0.30 | CGG-R | 2 | 0.05 |
| AUU-I | 302 | 8.21 | ACU-T | 85 | 2.31 | AAU-N | 112 | 3.05 | AGU-S | 32 | 0.87 |
| AUC-I | 28 | 0.76 | ACC-T | 16 | 0.44 | AAC-N | 21 | 0.57 | AGC-S | 6 | 0.16 |
| AUA-M | 199 | 5.41 | ACA-T | 74 | 2.01 | AAA-K | 81 | 2.20 | AGA-S | 70 | 1.90 |
| AUG-M | 24 | 0.65 | ACG-T | 5 | 0.14 | AAG-K | 15 | 0.41 | AGG-S | 0 | 0.00 |
| GUU-V | 95 | 2.58 | GCU-A | 103 | 2.80 | GAU-D | 57 | 1.55 | GGU-G | 85 | 2.31 |
| GUC-V | 9 | 0.24 | GCC-A | 23 | 0.63 | GAC-D | 15 | 0.41 | GGC-G | 3 | 0.08 |
| GUA-V | 104 | 2.83 | GCA-A | 62 | 1.69 | GAA-E | 74 | 2.01 | GGA-G | 119 | 3.24 |
| GUG-V | 14 | 0.38 | GCG-A | 2 | 0.05 | GAG-E | 18 | 0.49 | GGG-G | 28 | 0.76 |

Table S4. The assembled mitogenomes coverage distribution of *Striatobalanus tenuis*

| **Specie** | **Genome length** | **sd** | **Mean** | **2.5%coverage** | **97.5%coverage** | **Min** | **Max** |
| --- | --- | --- | --- | --- | --- | --- | --- |
| *Striatobalanus tenuis* | 15,067 | 8.02 | 30.13 | 15.00 | 45.00 | 2.00 | 54.00 |


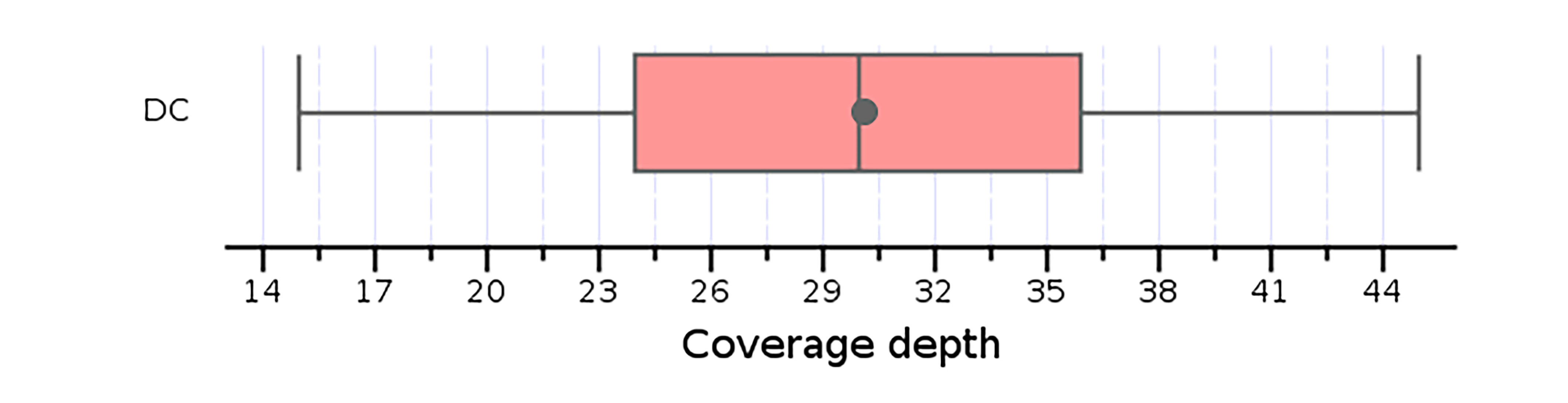


Figure S1. The assembled mitogenomes coverage depth of *Striatobalanus tenuis*
