# Supplementary material for: Complete mitochondrial genome of Striatobalanus tenuis Hoek, 1883 (Balanomorpha: Balanidae) and a novel molecular phylogeny within Cirripedia
Source: Mitochondrial DNA B Resour. 2024 Jan 3;9(1):29–32. doi: 10.1080/23802359.2023.2299087 (PMC10769117; doi:10.1080/23802359.2023.2299087)

DC: Coverage distribution

Id	N	Mean	Median	sd	q1	q3	2.5% percentile	97.5% percentile	Min	Max
DC	15,067	30.13	30.00	8.02	24.00	36.00	15.00	45.00	2.00	54.00

Histogram

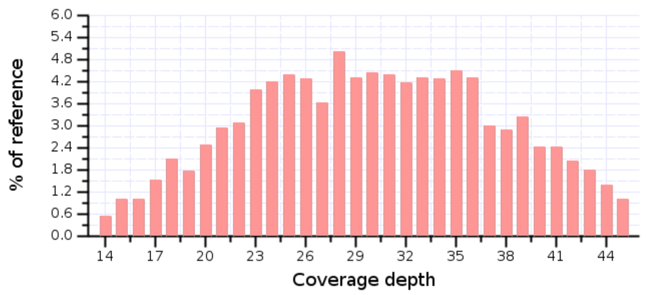

Cumulative histogram

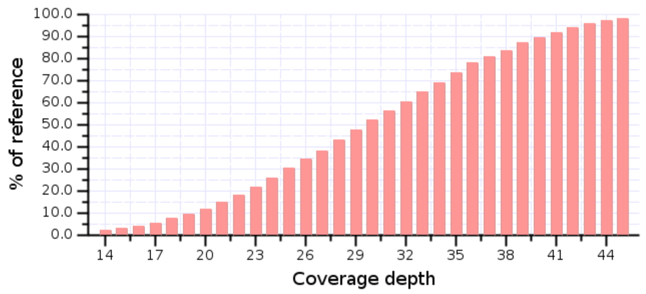

Box and Whisker summary

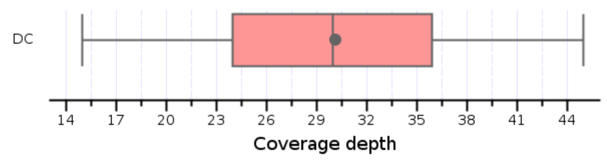

Supplement: Supplemental Material [file TMDN_A_2299087_SM5169.pdf]
